# Depression/Anxiety in Homebound Older Adults: Effects of Loneliness and Psychological Well-being

**DOI:** 10.1093/geroni/igaf122.2704

**Published:** 2025-12-31

**Authors:** Angelina Gutierrez, Kelly Vences, Brian Fons, Namkee Choi

**Affiliations:** The University of Texas School of Social Work, Austin, Texas, United States; The University of Texas School of Social Work, Austin, Texas, United States; The University of Texas at Austin, Austin, Texas, United States; The University of Texas at Austin, Austin, Texas, United States

## Abstract

The prevalence of depression/anxiety, loneliness, and psychological distress is much higher among homebound older adults than among their non-homebound peers. In this study, we examined the direct effect of a homebound state (defined as never/rarely going outside the home in the preceding month) on depressive/anxiety symptoms and the mediation effect of loneliness and psychological well-being (assessed with purpose in life, meaning in life, confidence, desire to improve life, and satisfaction with living situation) on the associations between homebound state and depressive/anxiety symptoms. Data came from the 2023 National Health and Aging Trend Study (N = 7,447 community-dwelling Medicare beneficiaries age 65+). Results from a path model showed that depressive/anxiety symptoms were positively associated with the homebound state (B = 0.78, SE = 0.22, t = 3.54, p=.001) and loneliness (B = 0.63, SE = 0.05, t = 12.59, p<.001), but negatively associated with psychological well-being (B=-0.43, SE = 0.03, t=-12.36, p<.001). Bootstrapped results show a significant indirect effect of a homebound state on depressive/anxiety symptoms through loneliness (0.13, 95% CI = [0.03, 0.24], z = 2.47, p=.014) and psychological well-being (0.28, 95% CI = [0.18, 0.37], z = 5.62, p<.001). The ratio of the indirect effect of loneliness on a homebound state to the total effect of a homebound state on depressive/anxiety symptoms (indirect effect [0.13] + direct effect [0.78]=0.92) was 0.15. The ratio of the indirect effect of psychological well-being on a homebound state to the total effect of a homebound state on depressive/anxiety symptoms (indirect effect [0.28] + direct effect [0.78]=1.06) was 0.26. Interventions to decrease loneliness and improve psychological well-being among homebound older adults are needed to alleviate their depression/anxiety.

